# Jasmonic Acid: An Essential Plant Hormone

**DOI:** 10.3390/ijms21041261

**Published:** 2020-02-13

**Authors:** Kenji Gomi

**Affiliations:** Plant Genome and Resource Research Center, Faculty of Agriculture, Kagawa University, Miki, Kagawa 761-0795, Japan; gomiken@ag.kagawa-u.ac.jp

The plant hormone jasmonic acid (JA) and its derivative, an amino acid conjugate of JA (jasmonoyl isoleucine: JA-Ile), are signaling compounds involved in the regulation of cellular defense and development in plants. The number of articles on JA has increased dramatically since the 1990s. JA was recognized as a stress hormone that regulates plant responses to biotic stresses, such as those elicited by hervivores and pathogens, as well as abiotic stresses, such as wounding and ultraviolet radiation. Recent studies have progressed remarkably in understanding the importance of JA in the life cycle of plants. It has been revealed that JA is directly involved in many physiological processes, including stamen growth, senescence, and root growth. Furthermore, it has been known to regulate the production of various metabolites, such as phytoalexins and terpenoids. Many active regulatory proteins involved in the JA signaling pathway have been identified by screening for *Arabidopsis* mutants. The discovery of the JA receptor, CORONATINE INSENSITIVE 1 (COI1), and the central repressors, jasmonate ZIM (JAZ)-domain proteins, further promotes the efforts to understand the JA signaling pathway in *Arabidopsis*. However, many aspects about the JA signaling pathway in other plant species remain to be elucidated.

This Special Issue, “Jasmonic Acid Pathway in Plants” contains five review articles published by field experts. Information available on the important role of JA in plant growth has been updated in these reviews [1,2,3,4,5]. These reviews will help in understanding the crucial roles of JA in its response to the several environmental stresses and developments in plants. In addition, this Special Issue also contains 15 original research articles. The noteworthy fact is that studies published in this Special Issue were performed using several plant species, including those belonging to *Arabidopsis*: *Camellia sinensis* [6], *Panax ginseng* [7], *Oryza sativa* L. [8,9], *Prunus avium* L. [10], *Brassica rapa* [11], *Sorghum bicolor* L. [12,13], *Nicotiana benthamiana* [14], *Pogostemon cablin* [15], *Zea mays* [16], and *Arabidopsis thaliana* [17,18,19,20]. This indicates that JA is an essential plant hormone across different plant species. Furthermore, these articles prove that JA has different roles during the vegetative and reproductive stages of plant growth. Gladman et al. [12] and Dampanabonia et al. [13] reported that JA negatively affects grain number in sorghum, suggesting that a new breeding approach that modifies JA-biosynthesis genes using genome editing can lead to increased grain yield in cereals. Below, I will focus on two studies published in this Special Issue as a plant pathologist.

Uji et al. demonstrated that the JA-induced VQ-motif-containing protein, OsVQ13, positively regulates the JA signaling pathway in rice [9]. Interestingly, OsVQ13 also activates the salicylic acid (SA) signaling pathway and confers resistance to rice bacterial blight, which is caused by the hemibiotrophic pathogen, *Xanthomonas oryzae* pv. *oryzae* (*Xoo*), in rice [9]. Generally, SA confers resistance to biotrophic and hemibiotrophic pathogens, whereas JA has been known to confer resistance to necrotrophic pathogens in plants. The relationship between JA and SA is antagonistic in many plant species. However, this antagonistic crosstalk between JA- and SA-dependent defense signaling pathways remains unclear in rice. It has been reported that a central repressor of the JA signaling pathway negatively affects *Xoo* resistance in rice [21]. JA-induced volatiles, such as monoterpenes, act as antibacterial or signaling compounds in the defense response against *Xoo* [22,23]. Uji et al. also demonstrated that the central positive regulators of the SA signaling pathway are induced by JA in rice [9]. It has also been suggested that OsVQ13 plays a critical role as an activator involved in both JA- and SA-induced resistance to *Xoo*. Taken together, these results strongly indicate that the JA and SA signaling pathways interact coordinately to yield induced defense responses in rice. Rice may develop a unique system to shield itself against pathogens. Recently, this unique system has been called “Common Defense System” [24].

Nakano and Mukaihara published an excellent study in this Special Issue [14]. The pathogen *Ralstonia solanacearum* is known to be hemibiotrophic and causes bacterial wilt disease in more than 200 plant species, such as tomato, potato, banana, and eggplant. Plants have developed a specialized defense system, the so-called pattern-triggered immunity (PTI), to inhibit or attenuate infection due to *R. solanacearum*. To suppress PTI, the pathogen injects approximately 70 type III effectors into the plant cells through the Hrp type III secretion system. Nakano and Mukaihara identified an effector, RipE1, which promoted the degradation of JAZ repressors and induced the expressions of JA-responsive genes in a cysteine–protease-activity-dependent manner [14]. JA and SA signaling pathways have been shown to antagonize each other in *N. benthamiana*. Thus, the activation of the JA signaling pathway causes the suppression of the SA signaling pathway, which is essential for the defense response against *R. solanacearum*. They also revealed that the effector RipAL induces JA production to activate its signaling pathway and simultaneously suppress the SA-mediated defense response in these plants [25]. These results indicate that *R. solanacearum* hijacks the JA signaling pathway and exploits antagonistic interactions between the JA and SA signaling pathways to promote a successful infection. They unraveled one of the survival strategies, which was previously unknown.

Finally, I would like to express my heartfelt gratitude to all of the authors and referees for their tremendous and relentless efforts in supporting this Special Issue. Without their valuable assistance, I would not have had an opportunity to publish this timely and successful publication, with its useful updates on the JA signaling pathway in plants. I also thank the assistant editor Sydney Tang for supporting my works.
